# Psychometric Properties of the Physical Activity Questionnaire for Older Children in Italy: Testing the Validity among a General and Clinical Pediatric Population

**DOI:** 10.1371/journal.pone.0156354

**Published:** 2016-05-26

**Authors:** Erica Gobbi, Catherine Elliot, Maurizio Varnier, Attilio Carraro

**Affiliations:** 1 Department of Biomedical Sciences, University of Padua, Padova, Italy; 2 Department of Sport, Exercise and Health, University of Basel, Basel, Switzerland; 3 Department of Medicine, University of Padua, Padova, Italy; Pondicherry Institute of Medical Sciences, INDIA

## Abstract

The purpose of this research was to assess an Italian version of the Physical Activity Questionnaire for Older Children (PAQ-C-It). Three separate studies were conducted, whereby testing general psychometric properties, construct validity, concurrent validity and the factor structure of the PAQ-C-It among general and clinical pediatric population. Study 1 (*n* = 1170) examined the psychometric properties, internal consistency, factor structure (exploratory factor analysis, EFA) and construct validity with enjoyment perception during physical activity. Study 2 (*n* = 59) reported on reliability, construct validity with enjoyment and BMI, and on cross-sectional concurrent validity with objectively measured MVPA (tri-axial accelerometry) over the span of seven consecutive days. Study 3 (*n* = 58) examined the PAQ-C-It reliability, construct validity with BMI and VO_2_max as the objective measurement among a population of children with congenital heart defects (CHD). In study 2 and 3, the factor structure of the PAQ-C-It was then re-examined with an EFA. The PAQ-C-It showed acceptable to good reliability (alpha .70 to .83). Results on construct validity showed moderate but significant association with enjoyment perception (*r* = .30 and .36), with BMI (*r* = -.30 and -.79 for CHD simple form), and with the VO_2_max (*r* = .55 for CHD simple form). Significant concurrent validity with the objectively measured MVPA was reported (rho = .30, *p* < .05). Findings of the EFA suggested a two-factor structure for the PAQ-C-It, with items 2, 3, and 4 contributing little to the total score. This study supports the PAQ-C-It as an appropriate instrument to assess the MVPA levels of Italian children, including children with simple forms of CHD. Support is given to the possible instrument effectiveness on a large international perspective in order to level out data gathering across the globe.

## Introduction

Physical activity (PA) is a leading health indicator and a key component for wellbeing among youngsters [1], however participation in PA dramatically declines as children grow into adolescents [2]. Public health actions are necessary to contrast this trend [3,4], and a sound understanding of childhood PA levels is fundamental in the progression of interdisciplinary health care and educational interventions. Measurement of habitual PA is a challenging task because PA is a complex behavior, there is no “one-size-fits-all” approach to data collection and analysis because of the variety of determinants, correlates and contextual variables that influence PA behavior [5]. Questionnaires have been described as “the only feasible method of assessing habitual physical activity in large populations” [6] because they are easy to administer, relatively inexpensive, and noninvasive [7]. On the contrary, objective measures would be most accurate, but involving higher cost, increased participant burden, and not providing information on activity type, location or context [8]. Questionnaires targeting children PA, must be simple and assist respondents with contextual or visual prompts [9]. Moreover, the psychometric properties of questionnaires must be solid in order to conduct theoretically-driven research and add substantive significance to findings.

The Physical Activity Questionnaire for Older Children (PAQ-C) [10] has been identified as a promising questionnaire by multiple studies as evidenced in a recent systematic literature review on PA questionnaires for children [11]. It has well-establish psychometric properties and desirable measurement characteristics in comparison with other self-report instruments for youth [11, 12]. Moreover, a calibration model, that allows raw PAQ-C scores to be converted in moderate to vigorous PA (MVPA) levels, has been recently developed [13]. The questionnaire is designed to assess general MVPA levels of school-aged children from grades four to eight. The PAQ-C was initially used in the Saskatchewan Pediatric Bone Mineral Accrual Study [14]. The instrument has seen further usage in a number of studies, including the Healthy Bones Studies [15] and “Action Schools! BC model” study [16].

The PAQ-C has been widely used in PA research among children aged 8 to 14, and it is specific related to the time of school year during which is completed (there may be differences in PA depending on seasonal period and for comparability issues it would be better to consider data collected in the same season). Its terminology is simple for children to understand and it is possible to administrate to groups as well as individuals. The PAQ-C is a 7-day recall instrument, it consists of 10 items and lastly scores by means of 9 items between 1 (low PA) and 5 (high PA). The first item is an activity checklist of common sports and games, the next six questions assess PA in specific moment of the previous week: physical education lesson, recess, lunch time, right after school, in the evening, and during the weekend. The eighth item asks which statements “describes you best for the last 7 days”, and the ninth item is a checklist on the frequency of PA over the previous week. The tenth question, not useful for the scoring, asks children if something prevents them from doing their regular PA.

The PAQ-C psychometric properties have been tested and reported as acceptable to good especially among white Canadian children [10, 17]. The resultant convergent validity of the questionnaire has been assessed against objectively measured PA by means of a Caltrac activity monitor (r = .39). More recently, the PAQ-C has elucidated a correlation with cardiorespiratory fitness (as measured by shuttle lap count) among boys and girls in the UK [18]. Observing the PAQ-C measurement properties in the UK, Thomas and Upton [19] reported good internal consistency (α = .82 and .84), a two-factor structure (broadly relating to PA “in school” and “outside of school”), a construct validity assessed by multiple regression analysis with the Self-Report Habit Index (SRHI; R^2^ change = .30), and a significant convergent validity with cardiovascular fitness measured by the ½ mile walk/run test (*r* = -.38). Moore and colleagues [20] investigated the psychometric properties of the questionnaire among children of different races. Results indicated that among white children, positive correlations existed between the PAQ-C and BMI (*r* = -.16) as well as PAQ-C and a step test (*r* = .30). Contrastingly, the PAQ-C showed no associations between these variables in relation to physical fitness among African American and Hispanic children. Moreover, after removing the item regarding PA during lunch, a two-factor structure has been suggested.

Furthermore, the PAQ-C has been indicated as a promising PA assessment in overweight and obese children [21] and appears to be an easy to use instrument aimed for use in large epidemiological investigations. However, a dearth of knowledge regarding the PAQ-C validity and reliability within Europe [20], with exception of the aforementioned UK studies [18,19], claims deeper psychometric exploration of the instrument. Methodological advances and validity in the field of children self-report PA measures could be of great importance in enhancing the understanding of the complex relations between PA levels and dietary habits, diseases risk, and body measures [22].

The purpose of the present investigation is to evaluate an Italian version of the PAQ-C (PAQ-C-It). Namely, the present paper aims to provide evidence regarding the validity of this questionnaire by evaluating general test score characteristics and factor structure, by providing validity evidence based on relations with other variables (construct validity) according to van Poppel and colleagues [23], and by testing the questionnaire in a clinical population. In order to accomplish this task, the Institutional Review Board of the University of Padua (Italy) for the protection of human subjects approved all forms and procedures of a large research protocol which includes three independent studies. First, study 1 evaluated the general test score characteristics, internal reliability, factor structure of the PAQ-C-It and construct validity with enjoyment perception during PA. Enjoyment has been recognized as one of the main reasons for participation in PA [24] thus providing evidence of validity and enhancing results by Moore and colleagues [20] that reported a positive convergent validity of *r* = .16 (*p* < .01). The enjoyment perception during PA has been considered a key factor in PA participation [24, 25] and consequently it has been hypothesized that children with high scores on the PACES would also report a high PAQ-C-It total score, and vice versa. The hypothesis of parallel scores on PACES and PAQ-C-It is supported by the notion that activities eliciting enjoyment or positive outcomes could instigate repeated or habitual participation in the activity [26]. Next, in study 2 concurrent validity of the PAQ-C-It with PA measured objectively by a tri-axial accelerometer has been cross-sectionally examined, BMI and enjoyment perception were selected to test the PAQ-C-It construct validity. Finally, study 3 reported on the psychometric properties of the questionnaire in a clinical setting involving children with a congenital heart defect (CHD). Given that children with CHD are encouraged by their general practitioner to be physically active, testing the PAQ-C-It in this population was deemed appropriate. In particular, questions arise as to how much PA these children are performing and whether they are meeting the PA guidelines [27–29]. To sufficiently answer these questions, PAQ-C-It validity has been tested in correlation with maximal oxygen consumption (VO_2_max) and BMI in children with CHD.

## Materials and Methods

### Ethics statement

The study was conducted according to the Declaration of Helsinki and is part of a larger research protocol. The Institutional Review Board of the University of Padua, Italy, approved the research protocol and the three studies presented in this paper. All participants and their parents provided written informed consent prior to their enrollment in the study 1, 2 and 3.

### Study 1

#### Participants

A convenience sample of 18 schools within the same district of northeastern Italy was involved in the study. Fourth and fifth grade students were invited to participate in the study and a total of 1,170 children returned both an assent and consent form signed in order to participate in the study. Thus, a total of 72 classrooms were involved (3 to 5 classrooms in each school) with 16 students per classroom on average (12 to 22 students per class). Participants were asked to complete a questionnaire package. Data were excluded from analysis if pupils indicated in the PAQ-C item 10 that they were unable to perform their normal PA during the previous week (as per the PAQ-C scoring manual), in addition to incomplete questionnaires. After this data cleaning, the final group considered for assessment consisted of 584 boys and 532 girls (*n* = 1,116) with a mean age of 9.3 ± 0.6 years.

#### Measure

Participants completed a questionnaire package, including demographic information, the PAQ-C-It (see procedure), and the Physical Activity Enjoyment Scale (PACES) [30, 31]. The PACES is a 16-item scale with ratings on a 5-point Likert type scale that ranges from 1 (“Disagree a lot”) to 5 (“Agree a lot”). Each sentence starts with, “When I am active …”, followed by nine positive items (e.g. “I enjoy it”, “I find it pleasurable”, “it gives me energy”), and seven negative items (e.g. “I feel bored”, “I dislike it”, “It’s no fun at all”). The sum scores range from 16 to 80, with higher scores corresponding to higher perceived enjoyment during PA.

#### Procedure

In order to conduct a first-step validation study of the PAQ-C in the Italian population, an Italian version of the PAQ-C (PAQ-C-It) was developed. According to the World Health Organization recommendations [32], two researchers independently translated the original PAQ-C into Italian and then a professional translator translated it back to English. Comparison of the differences between the original and the back-translated questionnaire was performed by the group of research, adapting and refining Italian version. To provide an appropriate cultural adaptation of the questionnaire, the PAQ-C-It differs from the original version in several ways. In particular, in the checklist of activities, in the first item, “in line skating”, “football”, “street hockey”, “floor hockey”, “cross country skiing”, and “ice hockey/ringette” were changed to “roller skating”, “rugby”, “hockey”, “tennis”, “karate/martial arts”, and “artistic/rhythmic gymnastics”, to better fit the Italian context.

A written informed consent was sent home with all students and only students who returned this form signed by a parent or guardian was allowed to participate. Questionnaires were distributed during a physical education class, thus allowing for completion during school hours in the spring season.

#### Statistical analysis

General test score characteristics of the PAQ-C-It were investigated. Descriptive analyses of items were examined separately for boys and girls as well as for the total group. Cronbach’s alpha was measured and corrected item-total correlations (CITs) were evaluated to report the item/scale relationship. Gender effect was investigated using a MANOVA and gender differences on the 9 items and on the PAQ-C-It total score were investigated by means of independent sample t-tests. Exploratory factor analysis (EFA) was conducted to explore the structure of the Italian version of the questionnaire, and construct validity has been tested by calculating the Pearson’s correlation between PACES and PAQ-C-It. Data were analyzed using SPSS 19, and significance level was set at *p* < .05.

#### Results

Descriptive statistics for PAQ-C-It items, by male, female, and the total sample are reported in Table 1. Scale reliabilities for girls (α = .75), boys (α = .72), and the total (α = .74) were acceptable. Mean CITs were .47 when considering the entire sample, .42 for boys and .47 for girls. A 2 x 9 (gender x items) MANOVA elucidated a significant gender effect (Wilks λ = .957; *p* < .001). Independent sample t-tests identified significant differences by gender on all items scores, with boys scoring higher than girls (*p* < .01, and *p* < .05 on item 4) on all items except for the first item (the checklist of activities) where boys and girls reported similar values. The independent sample t-test on the PAQ-C-It total score reported values significantly higher for boys than girls (*t* = 5.99, *p* < .001).

**Table 1:** Descriptive statistics for PAQ-C-It.

		Girls (*n* = 532) Mean (SD)	Boys (*n* = 584) Mean (SD)	All (N = 1116) Mean (SD)
**Checklist**	**Q1**	1.79 (.39)	1.82 (.41)	1.8 (.40)
**PE class**	**Q2**	3.84 (1.19)**	4.07 (1.08)	3.96 (1.13)
**Recess**	**Q3**	3.71 (1.18)**	4.03 (1.21)	3.88 (1.20)
**Lunch**	**Q4**	2.66 (1.39)*	2.87 (1.55)	2.77 (1.48)
**After school**	**Q5**	3.14 (1.15)**	3.44 (1.21)	3.30 (1.19)
**Evenings**	**Q6**	2.47 (1.21)**	2.70 (1.32)	2.59 (1.27)
**Weekend**	**Q7**	2.81 (1.26)**	3.05 (1.35)	2.94 (1.31)
**Description**	**Q8**	2.94 (1.24)**	3.28 (1.27)	3.12 (1.27)
**Week Summary**	**Q9**	2.99 (.91)**	3.22 (.97)	3.11 (.95)
**PAQ-C-It**	**total**	2.93 (.66)	3.12 (.66)	3.05 (.67)

PAQ-C-It mean values on items and total score, by gender and in total, for study 1.

* p < .05;

** p < .01 at independent sample t-test for gender differences.

The EFA was carried out employing Principal Component Analysis and Direct Oblimin Rotation on the data of the PAQ-C-It. Eigenvalues greater than 1 were reported for two factors, which accounted for 49.90% of the variance. Factor two was composed by item 2 (physical education), item 3 (PA at recess), and item 4 (PA at lunch), and all other items pertained to factor 1 (S1 File). Construct validity was evidenced by a significant Pearson’s correlation between the PAQ-C-It and the PACES (mean score = 69.6 ± 8.6, α = .84) at *p* < .001 (*r* = .36).

### Study 2

#### Participants

A convenience sample of 4^th^ and 5^th^ grade children from one school in the northeastern part of Italy was involved in the study (the school was not involved in study 1, but from the same district). A total of 59 children from 4 classrooms returned the informed consent signed by their parents, agreeing to participate in the second data collection. They wore a tri-axial accelerometer for 7 consecutive days and completed questionnaires after this week of objective measurement. Pupils who did not reach the valid wear time (see procedure section), or pupils who indicated that they did not perform their normal PA during the previous week in the PAQ-C-It, were excluded from analysis. The final group considered for the analyses consisted of 55 pupils (27 boys, 28 girls), one child was excluded because reported “yes” on question 10, another 3 children did not reach the valid accelerometer wear time. The participants’ mean age was 9.5 years (SD = 0.4 years).

#### Measures

Child weight (accurate to 0.5 Kg) and height (accurate to 0.1 cm) were recorded using a digital scale and a wall stadiometer (Seca Corporation, Hanover, MD). Height and weight were used to calculate BMI. To objectively evaluate habitual PA, a tri-axial accelerometer (GT3X+, ActiGraph, Pensacola, FL) was worn for 7 consecutive days. The same questionnaire package used in Study 1, comprising demographic information, the PAQ-C-It, and the PACES, was administered.

#### Procedure

One week prior to wearing accelerometers, weight and height were measured, with children wearing light clothing, and without shoes. Afterwards, pupils were instructed to wear the accelerometer continuously during the day for 7 consecutive days, except while sleeping, bathing, or swimming. Accelerometers were worn on the waist above the right hip using an elastic belt. Upon return of the GT3X+ unit, ActiGraph proprietary software (ActiLife Version 5.10) was used to download and convert the raw acceleration data into activity counts per 15 seconds (epoch). Data were analyzed in order to validate wear time and establish time spent in MVPA.

Non-wear time was defined as intervals with at least 60 consecutive minutes of zero counts. Counts were classified into the different PA intensity categories using the cut-points developed by Evenson and colleagues [33] that seemed the most accurately available ActiGraph cut-points for youth [34]. A day was considered a “valid monitoring day” when daily wear time exceeded 9 hours. At the end of the GT3X+ wearing period, participants were asked to complete the questionnaire by means of a group administration.

Parents received all information about the study in advance and were asked to accept or decline their child’s participation via their signature on a consent waiver. Only students who returned this waiver signed by a parent or guardian were allowed to participate.

#### Statistical analysis

General test score characteristics of the Italian version of the PAQ-C-It were investigated. Descriptive analyses of items were examined separately for boys and girls as well as for the total group. A cross validation of factor structure reported in study 1 was conducted with an EFA. Spearman correlation was conducted between PAQ-C-It total score and data on MVPA assessed by the GT3X+ accelerometers to test concurrent validity. Pearson’s correlation between PAQ-C-It and PACES and BMI was examined to provide construct validity. Significance level was set at *p* < .05.

#### Results

Descriptive statistics for all investigated variables are reported in Table 2. Scale reliabilities for girls (α = .72), boys (α = .75) and total group (α = .73) were acceptable. Independent sample t-tests indicated no significant gender differences in the PAQ-C-It items and the total score.

**Table 2:** Descriptive statistics for PAQ-C-It items, BMI and objective MVPA.

	Mean	SD
**Checklist Q1**	1.83	.36
**PE class Q2**	2.56	1.86
**Recess Q3**	4.51	.79
**Lunch Q4**	2.6	1.42
**After school Q5**	3.13	1.14
**Evenings Q6**	2.13	1.1
**Weekend Q7**	2.36	1.22
**Description Q8**	2.89	1.07
**Week Summary Q9**	3.07	.87
**PAQ-C-It total**	2.79	.52
**PACES**	71.9	6.3
**BMI**	17.9	2.3
**Objective MVPA**	249.8	82.9

Mean values of PAQ-C-It total score, PACES, BMI, and objectively measured MVPA in study 2 (*n* = 55). Objective MVPA is expressed as time in minutes spent in > 1399 counts per minute.

The cross validation of the PAQ-C-It was performed using an EFA. Specifically, Principal Component Analysis and Direct Oblimin Rotation on the PAQ-C-It data were calculated. Eigenvalues greater than 1 were reported for two factors, which accounted for 59.84% of the variance. In contrast with study 1, factor two was composed of item 2 and 4 (physical education and PA at lunch, respectively), with all other items pertaining to factor 1 (S1 File).

Concurrent validity was measured by correlating the PAQ-C-It total score with total MVPA measured by the tri-axial accelerometer. The PAQ-C-It total score significantly correlated with total objective MVPA (rho = .30), supporting that the questionnaire sufficiently measures the MVPA during a school week. Pearson’s correlation to test construct validity of the PAQ-C-It showed significance in both the association with the PACES (*r* = .30) and the association with BMI (*r* = -.30).

### Study 3

#### Participants

A convenience sample of parents or guardians of 76 patients with congenital heart defect (CHD) from the Complex Operative Unit for Sports Medicine at the University Hospital in Padua (Italy) ranging from 8 to 14 years old were contacted and invited to participate in the study. Parents and guardians received all information about the study and were asked to accept or decline their child’s participation via their signature on an informed consent waiver. Only patients who returned this form signed by a parent or guardian were allowed to participate.

Patients were not contacted if they had cardiac surgery in the last 6 months, or were restricted from participating in PA or physical education. Parental consents and the agreement of 58 children (34 boys and 24 girls, mean age = 11.6 ± 1.9 years) with CHD were obtained. In accordance with the functional classification of Connelly et al. [35], participants were considered as two separate groups depending on the complexity of the CHD. Specifically, simple forms of CHD correspond to congenital defects that need no treatment or are easily surgically treated (e.g. ventricular septal defect, atrial septal defect, coarctation of the aorta, pulmonary stenosis). Complex forms of CHD consist in more complex heart defects that required advanced surgically corrections (e.g. Tetralogy of Fallot, pulmonary atresia, transposition of the great vessels, Fontan circulation). The simple form of CHD group (SF) constituted 21 children (boys = 14, girls = 7), and the complex form of CHD group (CF) included 37 children (boys = 20, girls = 17). Within the CF group, children were further subdivided into two smaller groups, depending on functional class according to the New York Heart Association classification (NYHA) [36]. Subsequently, 18 children were classified into NYHA class I (CF1) and 19 in NYHA class II (CF2).

#### Measures

Participants completed the PAQ-C-It during a testing session at the hospital. Weight (accurate to 0.5 Kg) and height (accurate to 0.1 cm) were recorded using a digital scale and a wall stadiometer (Seca Corporation, Hanover, MD). Exercise capacity was evaluated by a cardiopulmonary maximal test, with direct evaluation using the VO_2_max.

#### Procedure

Participants were asked to participate in an individual testing session at the hospital. The first half of the session comprised the questionnaire administration, and height and weight measurements. In the second half, exercise capacity was evaluated with a VO_2_max treadmill test utilizing a ramped “breath by breath” gas analysis protocol.

Before starting the maximal exercise capacity evaluation, children received a detailed explanation of the testing procedure and its purpose, including the nature of the progressive exercise protocol, symptoms (heavy breathing, sweating) and possible complications. A resting supine standard 12-lead ECG was obtained preceding exercise (Case 8000 version 4.04, Marquette Electronics ine^™^). Afterwards, children were prepared for the treadmill test by fitting the face mask appropriately for the ventilatory expired gas analysis (CSD/Net System 2001, Medical Graphics Corporation^™^). The Bruce test with a ramp protocol was administered [37]. It comprised an ECG–monitored maximal exercise testing in which the child exercises to complete exhaustion as the treadmill speed and incline progressively increases. During the test, ECG, blood pressure and ratings of perceived exertion were also collected. The test finished when the rate of perceived exertion corresponded to a score between 18–20 on the Borg scale. The aforementioned cardiopulmonary exercise testing is supported as the most accurate noninvasive quantification of maximal aerobic capacity (i.e. VO_2_max) [37].

#### Statistical analysis

General test score characteristics of the Italian version of the PAQ-C-It among children with CHD were investigated. Descriptive analyses of items were examined separately by gender and by NYHA functional classification by performing a one-way ANOVA and a Bonferroni post hoc to evaluate possible differences. A cross-validation of factor structure reported in study 1 has been conducted by means of an EFA. Pearson’s correlation was conducted between the PAQ-C-It total score and the VO_2_max and BMI to test for construct validity.

#### Results

All 58 children completed the first half of the testing session (questionnaire and anthropometric measurements), yielding 100% adherence rate. This offered a sound sample size for descriptive statistics of the PAQ-C-It and BMI. Five children were either unable to complete the maximal test or they were not compliant with gas measurements and consequently, direct evaluation was not possible. Descriptive data for PAQ-C-It items, total score, age, BMI, and VO_2_max are showed in Table 3.

**Table 3:** Descriptive statistics for investigated variables in study 3, and ANOVA results by CHD group.

	SF (*n* = 21) Mean (SD)	CF1 (*n* = 18) Mean (SD)	CF2 (*n* = 19) Mean (SD)	*F*	*p*
**Checklist Q1**	1.74 (.32)	1.53 (.26)	1.70 (.36)	2.412	n.s.
**PE class Q2**	4.24 (.83)	3.17 (1.38)	3.68 (1.20)	4.242	< .05
**Recess Q3**	3.10 (1.22)	2.44 (.78)	2.95 (1.22)	1.801	n.s
**Lunch Q4**	2.33 (1.46)	1.39 (.70)	2.11 (1.20)	3.296	n.s
**After school Q5**	3.29 (1.01)	2.56 (.86)	2.63 (.89)	3.763	< .05
**Evenings Q6**	1.86 (.91)	1.39 (.78)	1.79 (1.32)	1.141	n.s
**Weekend Q7**	2.86 (.85)	1.72 (.75)	2.32 (1.0)	8.121	< .01
**Description Q8**	3.05 (.97)	2.33 (.77)	2.32 (1.0)	4.097	< .05
**Week Summary Q9**	3.03 (.84)	2.37 (.62)	2.61 (.94)	3.332	< .05
**PAQ-C-It Total score**	2.83 (.59)	2.10 (.28)	2.46 (.58)	9.997	< .001
**Age (years)**	11.3 (1.9)	12.2 (2.0)	11.4 (1.9)	1.270	n.s.
**BMI (kg/m**^**2**^**)**	17.7 (3.4)	19.2 (3.2)	17.0 (3.6)	2.001	n.s.
**VO**_**2**_**max (ml·Kg**^**-1**^**·min**^**-1**^**)**	(*n* = 20) 43.6 (7.9)	(*n* = 17) 41.1 (12.6)	(*n* = 16) 33.5 (6.2)	5.614	< .01

The PAQ-C-It reported moderate to good internal consistency, indicating α = .83 for girls, α = .70 for boys, and an α = .76 combined. Gender was neither significant in the PAQ-C-It items nor in the total score. The one-way ANOVA showed significant differences in the self-reported PA in the exercise capacity, when considering all three NYHA functional classifications (SF, CF1 and CF2) (F = 9.99, *p* < .001) (see Table 3). In particular, the post hoc analysis showed that the SF group reported significantly higher values for items 2, 5, 7, 9 and PAQ-C-It total score compared to the CF1 group. Exercise capacity, and PAQ-C-It item 9 (7-day summary) revealed significantly lower scores, or less weekly MVPA, in the CF2 group versus the SF group.

The cross-validation of the PAQ-C-It was performed with an EFA. Principal Component Analysis and Direct Oblimin Rotation on the PAQ-C-It data were selected so as to replicate the analyses of study 1 and 2. Eigenvalues greater than 1 were reported for two factors, explaining 57.16% of the variance. Mirroring study 1, factor two consisted of items 2, 3, 4 (physical education, school recess and PA at lunch), leaving all remaining items in factor 1 (S1 File).

Construct validity was assessed by correlating PAQ-C-It total score with the VO_2_max and BMI. Given that the complexity of the CHD had significantly determined different PAQ-C-It total scores, correlation analyses were performed separately for the two groups. Pearson’s correlation showed the self-reported PA to have a positive association with the maximal exercise capacity (*r* = 0.55, *p* < .01) and a negative association with BMI (*r* = -.79, *p* < .001) in the SF group. No significant correlations emerged for the PAQ-C-It total score among children in the CF group.

## Discussion

In the present study, psychometric properties of the Italian version of the PAQ-C [10] were tested to compile the results of three studies among Italian children with and without a clinical heart condition. Data on the PAQ-C-It for a total of 1229 children were reported providing several pieces of construct validity evidence based on the relation with other variables, including enjoyment, BMI, accelerometer scores. Only the EFA suggested a two-factor structure without a univocal solution in the three studies (Table 4).

**Table 4:** Summary of the findings from the three studies.

Population	Internal consistency	EFA results	Type of validity evidence	Validation variables
**Study 1**. Children from the general population (*n* = 1116)	α = .74	Factor 1 (item 1,5,6,7,8,9)	Construct validity	Enjoyment of PA (*r* = .36)
		Factor 2 (item 2,3,4)		
**Study 2.** Children from the general population (*n* = 55)	α = .73	Factor 1 (item 1,3,5,6,7,8,9)	Construct and concurrent validity	BMI (*r* = -.30)
		Factor 2 (item 2,4)		Enjoyment of PA (*r* = .30)
				Objective MVPA (rho = .30)
**Study 3.** Children with CHD (*n* = 58)	α = .76	Factor 1 (item 1,5,6,7,8,9)	Construct validity	BMI (*r* = -.79) for simple form CHD (*n* = 21)
		Factor 2 (item 2,3,4)		VO2max (*r* = 0.55) for simple form CHD (*n* = 21)

Internal consistency was investigated by reporting values of the Cronbach’s alpha. In general, the PAQ-C-It showed acceptable to good alpha values (α = .70 to .83) which accorded with the original results from Canada (.79 to .89), and also with more recent findings from the USA (α = .72 to .88) [38], and the UK (α = .82 to .84) [19].

Findings derived from the factor analyses conducted in the three studies suggested that the PAQ-C-It could have a 2-factor structure, particularly since items 2, 3 and 4 (PE, PA during recess, and PA at lunch respectively) did not contribute to the total score. The data could be further recoded into a factor referred to as “in school activity”, which comprised of items 2-3-4, and a factor referred to as “out of school activity”, which included all the other items. This dichotomization enabled a partial confirmation of the findings of Thomas and Upton [19] among English children and similar results have been found in other validation studies of the PAQ-C [20, 38]. However, a univocal factor structure for the PAQ-C is, at present, impossible to define. In our findings, item 3 (PA during recess) of the PAQ-C-It from study 2 did not correlate to the “in school” factor, which prevented a cross-validation of results from study 1 and study 3. This adds further support to Janz and colleagues [38], in that a modification of this item is necessary.

Construct validity was evaluated by correlating the PAQ-C-It total score with the PACES, the BMI, and with the VO2max for children with CHD. According to Cohen’s interpretation of effect size [39], the PAQ-C-It total score was moderately associated with the enjoyment perception registered by mean of the PACES-It, with *r* values ranging from .30 to .36. These results are enhanced by the findings of construct validity with BMI. Specifically, in the non-clinical group a correlation of *r* = -.30 was reported, in contrast to other studies in which these values among a non-clinical population were equal to -.16 [20] or were not significant [19]; within the SF group, a strong correlation of *r* = -.79 was found for the PAQ-C-It and the BMI. Despite the low sample size has to be considered, these findings allude to the notion that PA habits of children with the complex form of CHD are more likely to be influenced by the clinical condition itself more so than their BMI. Correlation of the PAQ-C-It total score with the maximal exercise capacity (VO_2_max) showed a coefficient of *r* = .55 in the SF group. Results from the total CF group were not significant even with regard to construct validity for the PAQ-C-It with the VO_2_max. It is thus advised to use caution in administering the questionnaire to children with a complex form of CHD, even though the reliability analysis showed good coefficient for the total CHD group (α = .76).

Finally, concurrent validity with the objectively measured PA during 7 consecutive days was evaluated. In our group of 55 children, the PAQ-C-It total score was significantly related to total MVPA as registered by the tri-axial accelerometer (rho = .30, p < .05). These findings replicate those of Kowlaski and colleagues [17], which reported a coefficient of r = .39 using Caltrac activity monitors. As such, these findings maintain that the PAQ-C is sufficient for assessing the habitual MVPA in school-aged children during their time spent in school. The Italian version of the questionnaire showed good concurrent validity given that a comparison with 7 days of objective monitoring has been conducted. This wavered from findings by Janz and colleagues [38] who reported higher concurrent validity (rho = .63) for the PAQ-A, an adolescent version of the questionnaire, but using 3 to 5 days of monitoring.

Limitations to the present study include the lack of test-retest reliability of the PAQ-C-It, and the lack of representation of different ethnicities (all participants were Caucasian and of Italian descent). Secondly, due to the non-random sampling method used in the three studies, a generalization of the results could not be achieved properly, however the characteristics of participants help identifying the population to which the findings might apply in Italy. Lastly, the low sample size of study 2 and 3 could have affected the results of the presented factorial analyses.

This study supports that the PAQ-C is an appropriate instrument to assess habitual MVPA in children in Italy and in particular, those with a simple form of CHD. It could be used for further large population studies on child PA or to measure MVPA as a confounding variable. Our findings, in general, showed moderate to good internal consistency, and moderate concurrent validity with MVPA as measured by accelerometry. Construct validity with VO_2_max, BMI, and with the PACES provided additional evidence in support of this Italian version of the questionnaire. Further research is necessary to confirm the validity and reliability of the questionnaire in test-retest administrations and in seasonal comparisons.

## Supporting Information

S1 File Results from EFA analyses in study 1, 2 and 3 and descriptive statistics for variables in the three studies. (DOCX)
