# Supplementary material for: Psychometric Properties of the Physical Activity Questionnaire for Older Children in Italy: Testing the Validity among a General and Clinical Pediatric Population
Source: PLoS One. 2016 May 26;11(5):e0156354. doi: 10.1371/journal.pone.0156354 (PMC4881960; doi:10.1371/journal.pone.0156354)
Supplement: S1 File — (DOCX) [file pone.0156354.s001.docx]

**Results of the EFA analysis for the three studies.**

The cross-validation of the PAQ-C-It was performed with an EFA. Principal Component Analysis and Direct Oblimin Rotation on the PAQ-C-It data were selected and eigenvalues greater than 1 were considered.

| **PAQ-C-It** | **EFA Study 1** | | **EFA Study 2** | | **EFA Study 3** | |
| --- | --- | --- | --- | --- | --- | --- |
|  | **Factor 1** | **Factor 2** | **Factor 1** | **Factor 2** | **Factor 1** | **Factor 2** |
| **Checklist Q1** | **.51** | .26 | **.82** | - .01 | **.61** | .33 |
| **PE class Q2** | .15 | **.38** | - .11 | **.84** | - .12 | **.76** |
| **Recess Q3** | - .03 | **.75** | **.52** | .23 | .49 | **.74** |
| **Lunch Q4** | - .70 | **.73** | - .40 | **.65** | .14 | **.50** |
| **After school Q5** | **.76** | - .13 | **.65** | . 36 | **.71** | .14 |
| **Evenings Q6** | **.73** | - .10 | **.66** | - .30 | **.60** | - .22 |
| **Weekend Q7** | **.69** | .04 | **.78** | . 27 | **.79** | .08 |
| **Description Q8** | **.75** | .09 | **.86** | . 32 | **.90** | - .14 |
| **Week Summary Q9** | **.78** | .05 | **.87** | - .11 | **.74** | .22 |

**Descriptive statistics for variables in the three studies**

| **Study 1** | **N** | **Minimum** | **Maximum** | **Mean** | **SD** |
| --- | --- | --- | --- | --- | --- |
| PAQ-C-It | 1116 | 1,3 | 4,9 | 3,1 | 0,7 |
| PACES_TOT | 1116 | 26,0 | 80,0 | 69,7 | 8,5 |
|  |  |  |  |  |  |
| **Study 2** | **N** | **Minimum** | **Maximum** | **Mean** | **SD** |
| BMI | 55 | 14,3 | 24,1 | 17,9 | 2,3 |
| MVPA_tot | 55 | 117,4 | 520,4 | 249,8 | 82,9 |
| PACES_TOT | 55 | 47,0 | 80,0 | 71,9 | 6,3 |
| PAQ-C-It | 55 | 1,5 | 3,7 | 2,8 | 0,5 |
|  |  |  |  |  |  |
| **Study 3** | **N** | **Minimum** | **Maximum** | **Mean** | **SD** |
| BMI | 58 | 11,6 | 26,8 | 18,0 | 3,4 |
| VO2max ml/kg/min | 53 | 23,5 | 84,0 | 39,7 | 10,0 |
| PAQ-C-It | 58 | 1,3 | 3,9 | 2,5 | 0,6 |
